# Quantification of biophysical adaptation benefits from Climate-Smart Agriculture using a Bayesian Belief Network

**DOI:** 10.1038/srep06682

**Published:** 2014-10-20

**Authors:** Patrick J. de Nijs, Nicholas J. Berry, Geoff J. Wells, Dave S. Reay

**Affiliations:** 1 School of GeoSciences, University of Edinburgh

## Abstract

The need for smallholder farmers to adapt their practices to a changing climate is well recognised, particularly in Africa. The cost of adapting to climate change in Africa is estimated to be $20 to $30 billion per year, but the total amount pledged to finance adaptation falls significantly short of this requirement. The difficulty of assessing and monitoring when adaptation is achieved is one of the key barriers to the disbursement of performance-based adaptation finance. To demonstrate the potential of Bayesian Belief Networks for describing the impacts of specific activities on climate change resilience, we developed a simple model that incorporates climate projections, local environmental data, information from peer-reviewed literature and expert opinion to account for the adaptation benefits derived from Climate-Smart Agriculture activities in Malawi. This novel approach allows assessment of vulnerability to climate change under different land use activities and can be used to identify appropriate adaptation strategies and to quantify biophysical adaptation benefits from activities that are implemented. We suggest that multiple-indicator Bayesian Belief Network approaches can provide insights into adaptation planning for a wide range of applications and, if further explored, could be part of a set of important catalysts for the expansion of adaptation finance.

The cost of adapting to climate change in Africa is estimated to be $20 to $30 billion per year, but the total amount pledged to finance adaptation falls significantly short of this requirement^1^ and less than half of the pledged amount has been disbursed^2^. However, future climate scenarios suggest the impacts of climate change could result in yield decreases of up to 20% by 2050 in the majority of African nations^3^, and population increase is likely to exacerbate pressures on food security. Climate-smart agriculture (CSA) aims to achieve a “triple-win” by sustainably increasing productivity, building resilience and reducing greenhouse gas emissions. As such, it attempts to assist in the achievement of food security and green development goals simultaneously^4^. Demonstrating progress towards these goals will be necessary to secure finance and plan interventions^5^. Without widely accepted frameworks to account for CSA adaptation benefits, performance-based finance is unlikely to make a significant contribution to the amounts required^6^,^7^,^8^. Demonstrating additionality in CSA adaptation finance^9^, by identifying where funding can be directed to increase resilience to future climate change rather than to current climates, remains an especially difficult undertaking. Models that integrate future climate scenarios with biophysical resilience assessments could therefore be of particular value.

Tracking changes to yields provides a straightforward method for assessing impacts on productivity, and mitigation benefits from CSA can be estimated by quantifying changes in stocks of carbon and net greenhouse gas fluxes associated with agricultural activity^10^. Measuring an increase in climate change resilience is more complicated, however, since adaptation benefits are influenced by a range of interacting factors that are realised over multi-annual or decadal timescales, and are highly context-specific^8^. There is therefore a need for frameworks that can track adaptation performance, assess opportunities and monitor any increase in resilience that is achieved^11^.

When planning for adaptation, an initial requirement is to assess whether the system is vulnerable to projected climatic changes. This vulnerability is defined by the system's exposure and sensitivity to climate change, and the system's capacity to cope with its effects^12^. Adaptation is achieved by improving *adaptive capacity* (defined as: ability to adjust, modify or change its characteristics to moderate potential damage^13^), or by reducing sensitivity and exposure to adverse impacts^14^. An understanding of biophysical and socioeconomic vulnerabilities at a local level is therefore needed to design activities that improve adaptive capacity or reduce sensitivity and exposure to the adverse impacts of climate change. Here we focus on understanding the impacts of adaptation activities on biophysical vulnerability, although the approaches used are equally suited to describing impacts on socioeconomic factors.

The exact form of these adaptation activities may be difficult to define and usually requires working with local stakeholders to identify key vulnerabilities to existing climatic conditions. Despite the existence of myriad frameworks that define the process of adaptation, many provide recommendations that are difficult to implement in low-income households where significant trade-offs between short and long term priorities may exist^15^,^16^. Given the relative lack of resources in the smallholder agriculture context, broad-brush frameworks can be seen as largely conceptual until best-practice solutions are identified in the field^17^. A re-usable framework that can efficiently model common impacts and capture local contexts simultaneously will not only be time and cost-effective, but could also generate information for investors within the timescales that most businesses operate, significantly aiding financial decision-making as a result^18^. While it is not argued that finance remains the only barrier to smallholder adaptation, the investment in adaptation that is urgently required might be more easily mobilised if the benefits of adaptation can be statistically captured^18^. As suggested here, a Bayesian Belief Network (BBN) approach can provide such a framework.

BBNs are increasingly being used in ecological modelling^19^,^20^,^21^. The BBN approach describes the probability of an outcome by considering the process that leads to that event, while taking account of the state of information describing the process^22^. Subjective probabilities are assigned to express a degree of belief in events occurring. The probabilities reported from BBNs are therefore considered as the degree of belief in any given outcome^23^ and allow for an estimation of the uncertainties attached to a process and its outcomes, especially when supporting data is sparse. This builds on the principle that useful probabilities need not necessarily be exact. Bayesian models are therefore quali-quantitative models, informed by expert judgment and continuously updated based on our best knowledge^24^. Most importantly, Bayesian models provide a framework in which decision-makers can input their current knowledge about a variable and its states, and assess the implications for the rest of the system conditional to it.

A BBN is often represented as an acyclic graph describing a network with a specified direction of flow between nodes. This means that basic BBNs cannot perform feedback loops^25^. When applied in ecological modelling, this characteristic can be viewed as both a strength and weakness. As ecological processes often possess feedback loops, these are typically excluded when modelling basic BBNs (although feedback loops can be included in more complex models^23^). Excluding feedback loops can help to focus the model on specific processes, however. This can be of value when managing complex ecological processes, particularly when decisions must be based on sparse data, and input from a wide variety of stakeholders^26^. Climate change adaptation involves a diverse range of processes, many of which are poorly understood. The management of climate change adaption is therefore well suited to the type of information provided by BBN applications.

Existing climate change adaptation evaluation frameworks make use of indicators that contain information about the effectiveness of adaptive actions^27^. Recent frameworks use either process-based indicators that consider impacts on adaptive capacity, or outcome-based indicators^28^ that describe overall vulnerability to climate change^22^. Both types of framework require the physical measurement of adaptation impacts over multi-annual timeframes, which is unlikely to be feasible in the smallholder agriculture context. To provide information relevant to smallholder agriculture, accounting frameworks should also be flexible enough to allow for the continuous integration of any site- and context-specific data^29^ made available via existing databases^30^.

The BBN we developed has two main modules (Figure 1); a process-based climate impacts assessment dependent on projected climate data and site specific-variables, and a vulnerability assessment module providing the output variables of exposure and sensitivity to climate change. This modular structure allows for the creation of a baseline against which the effectiveness of a set of agricultural interventions is tested. The interventions we considered are common in the CSA-context, for example intercropping (growing two or more crops in close proximity, providing each other with mutual benefits^31^), alley cropping (where crops are planted between alleys of trees that provide nutritional, and water availability benefits^32^), and legume fallows (rotation of nitrogen-fixing legumes with other crops^33^). These interventions are deemed to be low-regret options and are particularly appropriate to be assessed in the absence of detailed information concerning the site in question.

The agricultural interventions tested either reinforce the resilience of a particular site to climate impacts, or mitigate the modelled climate impacts directly. When introduced into the model, the agricultural interventions affect specific process variables and ultimately, the outcome-variable of vulnerability to climate change in terms of magnitude and probability. With this approach it is therefore possible not only to analyse which agricultural intervention, or a set of interventions, are most effective at lowering climate change vulnerability, but also to understand the reasons why. This trait is especially important where stakeholders can identify historic vulnerabilities to particular climate impacts and prioritise process-variables where interventions will provide the most effective adaptation outcome. Alternatively, they can identify which adaptation option has the most feasible capacity and resource requirements, and assess the costs and benefits of implementation. Importantly, it is also possible to identify cases of maladaptation, where action taken to reduce vulnerability to climate change would result in adverse effects^34^.

The dynamic nature of such a model also allows for exploring “what if”-scenarios. If a particular climate impact is likely to occur more frequently in the future, frequency of occurrence can be specified to explore the consequential impacts across the system. With this information it is possible to identify the most beneficial adaptation option in the scenario selected. Equally, if certain management practices will become more resource-efficient and prevalent in the future, how increased use of this practice would impact upon vulnerability to climate change in the future can also be modelled.

## Results

We applied the BBN model to assess the impacts of different agricultural interventions on biophysical vulnerability in Malawi based on projected 2060 climate data^40^. Malawi was chosen because of the significant risks to smallholder agriculture and the wider economy from future climate change in the region. Malawi's main staple crop is maize, which is grown on about 90% of cultivated land; tobacco is Malawi's main cash crop, accounting for about 60% of export earnings^35^.

**Figure 2:**
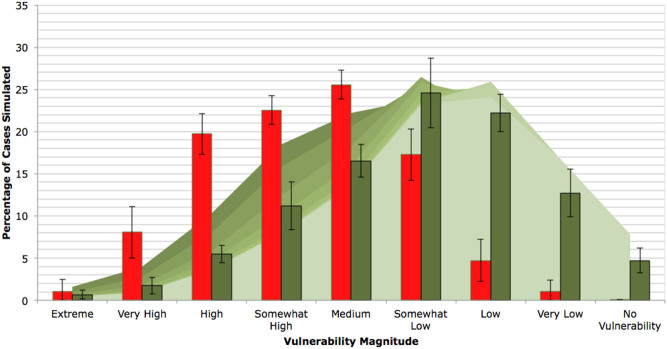
Malawi Smallholder Site Vulnerability to Climate Change. Displays magnitude of vulnerability to climate change as percentage of cases returned per 1000 cases simulated. The graph includes no-adaptation action baseline values in red (vulnerability assessment) shown as average percentage of cases per household based on a sample of 50 randomly selected smallholder farms in Malawi. Adaptation cases are shown in green shades, with increasing numbers of adaptation action in combination shown in lighter shades, based on 5 randomly selected smallholder farms (selected from the sample of 50). The mean of all adaptation combination cases are shown by the green bars. Error bars show the variation (standard deviation) between sites. See references 30,36–38 and methods section for data sources.

The vulnerability assessment baseline results presented in Figure 2 are based on 50 households randomly selected from a total sample of 12,271 households for which relevant data were available^30^.

The efficacy of the adaptation actions modelled is demonstrated by the shift from a frequency distribution for the no-adaptation baseline values (red bars in Figure 2) that peaks at *Medium* to *Somewhat High* vulnerability, to a frequency distribution for scenarios with adaptation actions (green bars in Figure 2) that peaks at *Somewhat Low* to *Low* vulnerability.

The cause of variability of mean vulnerability to climate change and mean biophysical adaptation benefits between sites is a direct consequence of the differences in site-specific biophysical conditions. Since constant values of national climate data^36^ were used for all scenarios, the differences of values observed between sites are a result of local variation in biophysical conditions, for example erosion proneness, slope or soil type.

**Figure 3:**
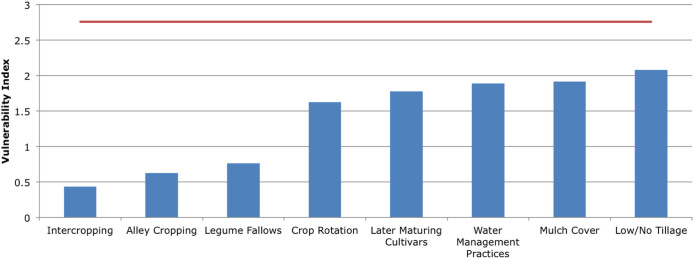
Single Adaptive Action Benefits. The vulnerability index (“VI”) is a function of probability and magnitude of simulated cases, centered on *Medium Vulnerability (Medium Vulnerability = 1*) and weighed at the extreme ends of the scales (see methods). The no-adaptation vulnerability index baseline is displayed in red, with single adaptation action VI values displayed in blue. These results are based on a sample of 5 randomly selected smallholder farms. See references 30,36–38 and methods section for data sources, and the Supplementary Information for a description of cropping systems.

To compare the effectiveness of different interventions at specific sites in Malawi, a Vulnerability Index (VI) derived from climate change impact probability and magnitude information was devised. The VI, informed by the BBN's process variables, is centered on the value of 1, which constitutes *Medium Vulnerability*. To demonstrate how the VI can be used at a specific site Figure 3 shows which single intervention is expected to increase biophysical resilience, and the related VI value. *Intercropping*, *Legume Fallows*, and *Alley Cropping* gave the greatest reduction in vulnerability by alleviating the negative climate impacts of decreased *Water Availability* and increased *Pests*. Specific case studies can be replicated for any site.

## Discussion

By calculating the impact of agricultural interventions on biophysical vulnerability from the BBN's probability and magnitude output, it is possible to directly quantify adaptation benefits. These could then be integrated into cost-benefit tools that serve to inform a quantitative investment analysis. Though difficulties remain in calculating monetary conversion factors for adaptation benefits defined in this network, this approach provides a starting point for comparing the relative impacts of different interventions and for assessing change with a standardised and transparent index. The Vulnerability Index (VI) described here expresses both the probability and magnitude of expected biophysical sensitivity to climate change. By interpreting how individual or combined adaptation actions affect the VI, it is possible to gauge an adaptation action's effectiveness in contributing to climate change resilience.

Increasing the number of adaptation actions increases total biophysical adaptation benefits (see Figure 2). However, returns on biophysical adaptation benefits gained per adaptation action diminish as adaptation actions are added to the model. The BBN outputs suggest that, of the interventions investigated, intercropping was the best approach to mitigate the climate impacts of decreased water availability and increased pests. This demonstrates the potential of the BBN framework for comparing adaptation approaches at a local level. The results also highlight the potential for using a BBN framework with a broader range of interventions, and enough sites to represent the variability over a larger area, to draw more widely applicable conclusions about the relative merits of different approaches.

Our work suggests that BBNs can be used to assess the impacts of CSA activities on biophysical vulnerability and determine which activities are appropriate to the site in question. Although the model outputs are developed from subjective decisions, these can be informed by expert collaboration to ensure probabilities reflect the best available information and understanding. Effective collaboration that uses triangulation or other consensus building approaches would reduce individual subjectivity when deciding on threshold values or designing subnets within the network. The BBN approach therefore provides a framework for making best use of current knowledge and is likely to be appropriate in a wide range of applications, particularly when resources for site-specific data collection are limited. Although the wide variety of data used, and subjective decision-making when defining variable relationships increase the uncertainty of our results, the model's flexibility allows it to be readily applied to identifying best-available adaptation practices at a broad range of sites. Only high-level data accessed from UN and World Bank databases were used in this study^30^,^37^,^38^. The use of national level climate projections adds a source of uncertainty to site specific outputs. Using local climate projection data, if available, would improve the BBN's climate impact estimates. However, the use of high-level data does allow for comparable assessments focusing on different sites and CSA activities.

The type of BBN used here can help address the current lack of adaptation benefit accounting tools by providing performance values to compare the expected impacts of adaptation activities. Given the great discrepancy between pledged and disbursed funds for adaptation^5^, potential for contributions from performance-based finance, and the food security, poverty reduction and disaster preparedness benefits of adaptation in the agriculture context, BBNs could provide a welcome addition to the climate change management toolbox. Not only could such an addition play a part in alleviating the lack of funds developing nations are experiencing in relation to their National Adaptation Programmes of Action^39^, it could also help advance progress towards Sustainable Development Goals, and the UN's first Millennium Development Goal of eradicating extreme poverty and hunger.

## Methods

The BBN is composed of five subnets. The network has been designed to capture the adaptation process, including the elements of climate change, climate impacts, local resilience and resulting vulnerabilities. Furthermore, a multi-variable binary-state adaptation subnet has been added that, when activated, will impact on either the climate impact or local resilience subnets. The return values without adaptation can therefore be compared against those with adaptation. A more detailed discussion of decision-making surrounding variable selection, data, interaction, type, and state with reference to applicable literature that informed the construction of the network and its underlying assumptions is presented in the Supplementary Information.

Subnet 1 includes a description of future climate projections based upon McSweeney *et al*.'s^36^ synthesis of the WCRP CMIP3 climate projection archive. These data have been selected to demonstrate that the model can be supported by data that are readily available. Having employed a consistent approach across 52 developing countries to provide an “off-the-shelf” analysis of climate data, the data provided are modelled by assuming conditions prescribed by the IPCC'S equally likely A2, A1B and B2 SRES^40^ scenarios. It details the projected 2060 climate differences as compared to Malawi's 1970–1999 baseline. The climate data are presented in quarterly-year blocks, running from December to February, March to May, June to August and September to November. For each of these reporting periods under each SRES scenario, a minimum (10^th^ percentile), median (50^th^ percentile) and maximum (90^th^ percentile) value is given and relating occurrence variables of the network reflect these. These data have been compiled into 3 individual nodes of the network, representing *Average Temperature, Average Precipitation* and *Extreme Precipitation* as per online availability of these data. To allow for a distinction between seasons, specific states representing the seasons of interest within these individual nodes can be selected to occur in 100% of the cases generated.

Subnet 2's variable network describes the impacts upon the site, informed by subnet 1 (climate change projections) and subnet 3 (site description). Geovariables of a randomly selected site in Malawi have been used for subnet 3, collected by the World Bank's “Integrated Survey on Agriculture”^30^,^37^,^38^. Of this dataset, 50 smallholder agriculture households were randomly selected from the total sample size of 12,271 to inform the vulnerability analysis. Of those 50 households, a further 5 have been randomly selected to inform adaptation option analysis. Variable relationships and associated subjective decision-making are defined by the cited literature sources (see Supplementary Information).

Subnet 4 details the adaptation options, with subnet 5 consisting of output variables. Intermediary nodes condense data of the previous subnets to feed into the output variable *Vulnerability to Climate Change* to reduce the strain of computing output variables. Subnet 5's resulting outcome-variable describes the site's vulnerability to climate change, whereas Subnets 1–3 describe the process-variables. As the operator of the network can change the input of adaptive action variables into the network, Subnet 4's variables are the control variables.

To aid the analysis of which adaptation option, or combination of adaptation options, lead to the highest reductions in overall probable vulnerability, a “*Vulnerability Index*” (“VI”) has been developed. The VI is a function of the *Vulnerability to Climate Change* (Subnet 5) output variable and its states (Equation 1): 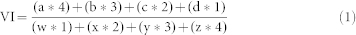
Where: *a = Extreme*

*b = Very High*

*c = High*

*d = Somewhat High*

*w = Somewhat Low*

*x = Low*

*y = Very Low*

*z = No vulnerability*

The VI's value is thus centered on the *Medium* state of the *Climate Change Vulnerability* variable. If VI > 1, vulnerability to climate change can be seen as high, if VI < 1, vulnerability to climate change can be seen as low. Therefore, the closer VI is to 0, the more effective the adaptation option was deemed to be in lowering vulnerability to climate change. This is the case with all threshold values within the network - subjective decisions have been made as to what constitute these thresholds (values are listed in Appendix B of the Supplementary Information). The VI is therefore an easy-to-use subjective index, informed by climate and site data, displaying the likely impact adaptation options have upon predefined thresholds of vulnerability to climate change.

Sensitivity analysis was used to assess which climate impacts were most responsible for higher vulnerabilities, and which site characteristics mostly affected these. A combinatoric approach was used to analyse which set of adaptation actions held most benefits and to assess whether some multi-adaptation responses hold less benefits than single-adaptation responses. The main source of uncertainty relates to subjective decision-making when designing variable interactions and magnitudes, along with the isolation of the described biophysical system to other, socio-economic system-impacting variables. It is not proposed that the model is ready for use in the field, instead it is used here to assess the feasibility of this approach for assessing adaptation performance. Expert collaboration to lower subjective bias in model design is essential for the BBN approach to be used in real-world applications.

## Author Contributions

P.J.D.N. designed the model, analysed the data and wrote the manuscript. N.B. contributed materials, analysis tools. D.S.R. and N.B. co-wrote the manuscript. G.W. undertook literature review and analysis.

## Supplementary Material

Supplementary Information Supplementary Information - Quantification of biophysical adaptation benefits from Climate-Smart Agriculture using a Bayesian Belief Network

## Figures and Tables

**Figure 1:**
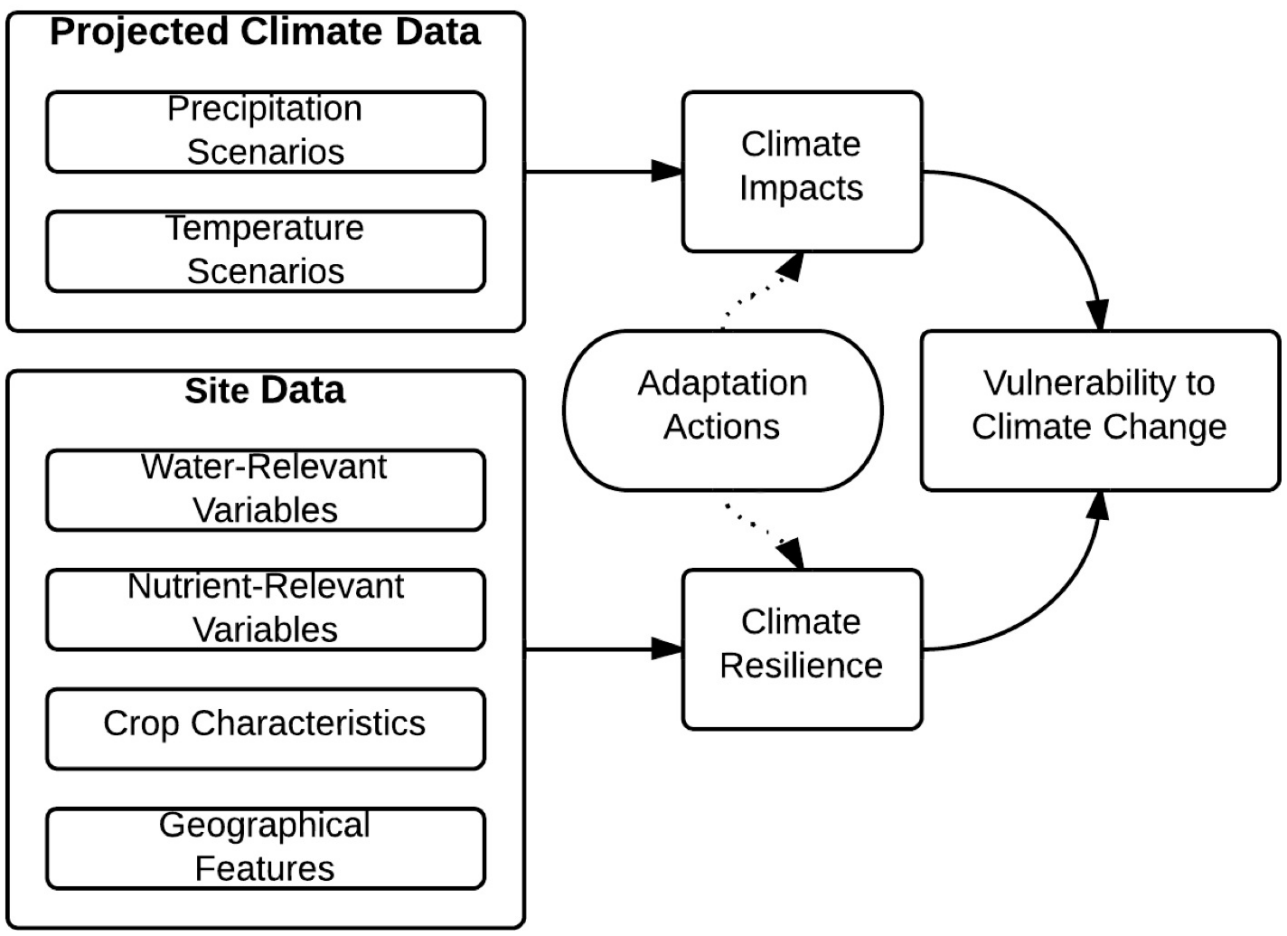
Structure of the Bayesian Belief Network. The conceptual diagram of the Bayesian Belief Network shows the elements of the impact assessment and vulnerability assessment. Adaptation actions are introduced to the vulnerability assessment and compared against the baseline no-action scenario. Site data and adaptation action variables are deterministic, with the rest of the model following stochastic principles defined by climate projection data scenarios in line with probabilistic occurrence ratios (defined in Supplementary Methods section). Variable relationships follow processes described in relevant literature (see Supplementary Information for references).
