# Supplementary material for: Quantification of biophysical adaptation benefits from Climate-Smart Agriculture using a Bayesian Belief Network
Source: Sci Rep. 2014 Oct 20;4:6682. doi: 10.1038/srep06682 (PMC4202202; doi:10.1038/srep06682)
Supplement: Supplementary Information — Quantification of biophysical adaptation benefits from Climate-Smart Agriculture using a Bayesian Belief Network [file srep06682-s1.pdf]

Supplementary Information - Quantification of biophysical adaptation benefits from Climate-Smart Agriculture using a Bayesian Belief Network

Patrick J. de Nijs*, Nicholas J. Berry, Geoff Wells, Dave S. Reay

School of GeoSciences, University of Edinburgh, Edinburgh EH8 9JX UK

email: patrick.denijs@gmail.com; david.reay@ed.ac.uk; nicholas.berry@ed.ac.uk; g.j.wells10@gmail.com

1. Network Design

Before describing the subnets of the Bayesian network in more detail, this section will explain its design principles, and the rationale behind them.

The modular structure is of the model allows for more flexibility and robustness (Merten, 2004: p.1). The network has been devised to capture the adaptation process as described in the literature, including the subnets of climate changes, climate impacts, local resilience and the resulting vulnerability. Furthermore, an adaptation subnet has been added that, when activated, will introduce positive changes to either the climate impact or local resilience subnets. As the network's return values without adaptation can therefore be compared with the values with adaptation, the integration of the adaptation subnet will serve the same purpose as in Musango and Peter's (2011) study where an "action"-output was compared with a "no-action"-output. Supplementary Figure 1 displays the design principle.

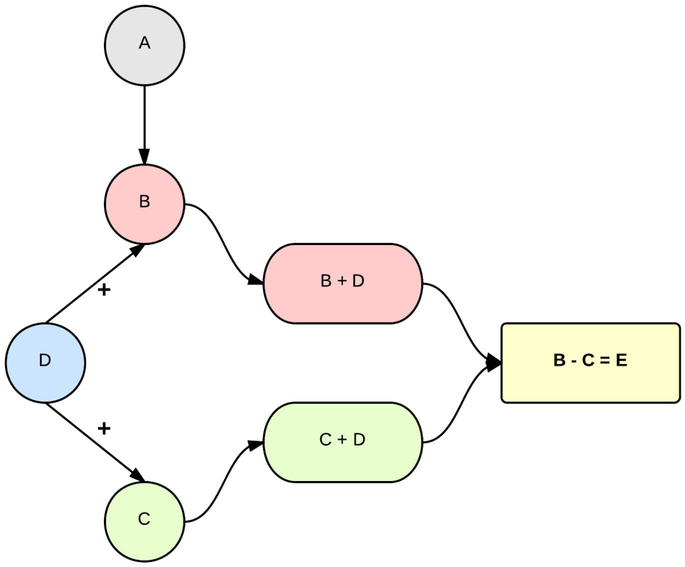

Supplementary Figure 1 – Design Principle of Bayesian Network in this study

The variables describe the following processes:

- *A = Climatic Changes*
- *B = Climate Impacts*
- *C = Local Resilience*
- *D = Adaptation Actions*
- *E = Vulnerability to Climate Change*

Therefore, this Bayesian network includes the following subnets:

- *Subnet 1:* A description of future climate conditions
- *Subnet 2:* The impacts of future climate upon the site of interest
- *Subnet 3:* A description of the characteristics of the site of interest
- *Subnet 4:* A set of adaptation options.
- *Subnet 5:* Vulnerability to climate change

Subnet 5's resulting outcome-variable describes the site's vulnerability to climate change. As the operator of the network can change the input of adaptive action variables into the network, *Subnet 4*'s variables are the control variables.

2.1 Description of the Process Variables (*Subnets 1-4)*

This section will detail the method and reasoning in which the *Subnets 1* to *4*, and their variables have been designed. It is structured into four parts, namely a basic subnet description, data source description, variable state description and variable link description. The basic description will provide information on which variables the subnet encompasses. The data source section will describe how the data used has been generated and collated, and will identify any limitations that are posed by using the data. Following that section, a justification of the number of variable states, their probabilities and the interaction with other variable states will be given, along with a definition of the terminology relating to each variable state. Lastly, a description will be given in how each variable affects the other in the variable link description section.

Each part will cover all four subnets before moving on to the next.

2.1.1 Basic subnet description

2.1.1. a) Subnet 1: A description of future climate conditions

The climate data subnet is composed of three variables deemed most significant for the agriculture sector (EPA, 2013). These are the following variables:

- Average Temperature (°C change as compared to baseline)
- Average Precipitation (mm/m^2 change as compared to baseline)
- Extreme Precipitation (% of rainfall falling in heavy events, change as compared to baseline)

2.1.1.b) Subnet 2: The impact of future climate upon the site of interest

This subnet describes the climate impacts identified as most significant in the IPCC's Fourth Assessment Report (AR4) on Africa (IPCC, 2007). The AR4 lists reductions in water availability, an increase in agriculture pests and diseases, erosion changes, and an increase of droughts in Sub-Saharan Africa. Given that different pests and diseases favour different climatic conditions and the associated difficulty of modelling those changes under a single variable, the most widespread pest affecting

maize production in Malawi has been selected ("African Stem Borer" (ASB); Biovision, 2011). Furthermore, building upon the AR4, Mueller *et al*. (2011) point to the changes to other important and related variables such as increases in evapotranspiration, soil temperature, plant heat stress and runoff. Therefore, as informed by those publications, the complete list of variables considered in the impact subnet is given below.

- Water availability (IPCC, 2007)
- Pest Spread: African Stem Borer (IPCC, 2007; Biovision, 2011)
- Erosion (IPCC, 2007)
- Drought (IPCC, 2007)
- Evapotranspiration (IPCC, 2007)
- Soil temperature (Mueller *et al.*, 2011)
- Plant heat stress (Mueller *et al.*, 2011)
- Runoff (Mueller *et al.*, 2011)

2.1.1. c) Subnet 3: A description of the characteristics of the site of interest

This subnet focuses on the site characteristics that are important in terms of defining resilience to climate change. To establish the network of variables that affects the site's resilience, Wells' (2012) framework of potential biophysical indicators has been used (Appendix A). The variables of interest that define biophysical resilience as compiled by Wells (2012) in a structured literature review, together with the references that support their significance to environmental resilience, are given below.

- Soil waterholding capacity (Tierfelder and Wall, 2009)
- Water use efficiency (Sinclair *et al.* 1984)
- Erosion control measures (Lal, 1974; Rishirumuhirwa, 1997)
- Root density (Banda *et al.* 1994)
- Soil nitrogen (Garba and Renard, 1991)
- Erosion Proneness (Obi and Nnabude, 1988)
- Floral diversity (Letourneau *et al.* 2011)
- Soil organic carbon (Banda *et al.* 1994)

Some of the variables identified by Wells have not been used, as they are described in the climate impacts subnet of the Bayesian network (e.g. *Evapotranspiration*). Also, due to data availability reasons, some variables have been

broken down into sub-components describing a similar process. These variables, and their impact upon variables identified by Wells (2012), are listed below.

- Crop temperature sensitivity (function of plant heat stress; Easterling and Agarval. 2007)
- Slope (function of erosion and runoff; Tierfelder and Wall, 2009)
- Soil type (function of soil waterholding capacity, water infiltration and runoff; Lal, 1974)

Due to the large amount of deterministic decision node variables in this subnet, there is a need to condense their information before impacting another subnet. This means that instead of information leaving the subnet through each individual node, it is bundled into a smaller number of variables (i.e. Nutrient Retention Capacity, Soil Nutrients). This is to limit the amount of resulting conditional probabilities of an individual node, which lowers computational demand.

2.1.1. d) Subnet 4: A set of adaptation options

The list of CSA adaptation options has also been informed by Wells' (2012) literature review resulting in the framework of potential biophysical indicators (Appendix A). The reason for this selection lies in the academically documented relationship of these adaptation options with resilience-building variables. The complete list of these options is given below.

- Mulch cover (Boli *et al.* 1993)

Covering the cropping area with mulch, improving the soil moisture conditions and limiting soil temperature fluctuations.

- Low/no tillage (Lal, 1974)

Refers to limiting disturbance of the top-soil, thereby improving soil rooting and moisture conditions.

- Crop rotation (Tierfelder and Wall, 2009)

Refers to the sequential planting of different crops, maintaining soil nutritional conditions and limiting impacts of pests.

- Intercropping (Chirva *et al.* 2007)

Growing two or more crops in close proximity, providing each other with mutual benefits

- Legume fallows (Sileshi and Mafongoya, 2006)

Rotation of nitrogen-fixing legumes with other crops, improving nutritional conditions of the soil.

- Alley cropping (Hulugalle and Kang, 1990)

Crops are planted between alleys of trees that provide nutritional, and water availability benefits.

- Later maturing cultivars (Tingem *et al.* 2009)

Refers to seeding crops later, or planting crops that naturally mature later. This seeks to alleviate adverse impacts of climate change (i.e. extreme temperatures) on crops during seasons traditionally for agricultural activities.

Furthermore, due to the lack of water management practices on the majority of farms in Malawi (FAO, 2006), a further adaptation practice has been introduced with positive effects upon water availability. Given the reliance upon rainfed agriculture in the majority of smallholder agricultures and relative availability of other sources of water in Malawi, we have specified an adaptation practice with minor positive effects on the variable of *Water Availability.* This practice seeks to emulate water efficiency gains through improved irrigation practices.

- Water management practices

2.1.2 Data Sources

2.1.2.a) Subnet 1: A description of future climate conditions

The climate projection subnet of the network has been designed by using McSweeney *et al.*'s (2010) synthesis of the WCRP CMIP3 climate projection archive. Having employed a consistent approach across 52 developing countries to provide an "off-the-shelf" analysis of climate data (McSweeney *et al.*, 2010), the data provided is modelled by assuming conditions prescribed by the IPCC'S equally likely A2, A1B and B2 SRES scenarios (IPCC, 2000). It details the projected climate differences as compared to Malawi's 1970-1999 baseline. The climate data is presented in quarterly-year blocks, running from December to February, March to May, June to August and September to November. For each of these reporting periods under each SRES scenario, a minimum (10th percentile), median (50th percentile) and maximum (90th percentile) value is given.

Three reporting years are provided, namely 2030, 2060 and 2090. This study will focus on the 2060 period, as climatic changes and their impacts have been identified to be significant enough to test adaptation responses while being close enough to limit the inherent uncertainties.

2.1.2. b) Subnet 2: The impact of future climate upon the site of interest

This subnet has received no direct data input. The value of its variables is defined by the climate data and adaptation option subnets.

2.1.2. c) Subnet 3: A description of the characteristics of the site of interest

The variables contained in this subnet are informed by the World Bank's "Integrated Survey on Agriculture" on Malawi ("LSMS-ISA: Third Integrated Household Survey"; World Bank, 2011). Using IBM SPSS software, data of fifty individual households was randomly selected to inform site characteristics outlined in Appendix B.

2.1.2. d) Subnet 4: A set of adaptation options

This subnet has received no direct data input. Their impact upon the model is described in section 3.1.5.

2.1.3 Variable Type

Bayesian networks can utilise different types of variables with different statistical properties. The Bayesian network software used (Netica) can define four different types of variables (or “nodes”), whereas only two types were used in this study (Norsys, 2013). Decision nodes describe variables that the operator can control. To make an impact upon the results generated, the operator will need to define the state of each decision node prior to running the Bayesian network. Nature nodes are basic variable nodes and can take two different forms. A nature node is deterministic when a set of parent combination leads to a state of the nature node in a deterministic way, while a probabilistic nature node is affected by its parent combinations in a likelihood fashion. This section will explain the variable type, and the reasoning why the type has been selected.

2.1.3 a) Subnet 1: A description of future climate conditions

The climate data subnet of the Bayesian Network uses probabilistic nature nodes. The minimum and maximum values are assigned each a 10% chance of occurring, while the median value is assigned an 80% chance of occurring. This is in line with the percentiles supplied in McSweeney *et al.* (2010).

2.1.3 b) Subnet 2: The impact of future climate upon the site of interest

The variables are defined by deterministic nodes. The reason for defining the climate-impact relationship in a deterministic fashion is one of necessity, as no study has currently assessed specific probabilistic values of each specific impact occurring in relation to climate projections in Sub-Saharan Africa. The deterministic relationship to the climate data means that specific climate data values lead to specific magnitudes of impact. The principle of this relationship is chosen to be as conservative as possible (i.e. the lowest-magnitude interaction possible) to mitigate positive bias (Druzdzel and Onisko, 2008).

2.1.3 c) Subnet 3: A description of the characteristics of the site of interest

These variables are defined by decision nodes. This relates to the principle that the site characteristics must be pre-selected by the operator before running the network.

2.1.3 d) Subnet 4: A set of adaptation options

These variables must also be decision nodes, as they are the control variables in the network.

2.1.4 Variable State Selection

2.1.4 a) Subnet 1: A description of future climate conditions

For each of the variables (*Average Temperature, Average Precipitation*, and *Heavy Rainfall Events*) 36 variable states have been defined. These states relate to values given in each 3-month reporting period, under each SRES scenario considered with all three (minimum, median and maximum) values included (Supplementary Figure 2). The strength of including all of the values into the same variable rather than creating multiple variables lies with the ability to instruct the Bayesian network only to investigate a specific SRES scenario or, specific reporting periods. Especially when considering maturing or harvesting periods where vulnerability to potential impacts would be especially high, this characteristic would allow the operator to identify climate impacts posed in periods of this high strategic vulnerability. Furthermore, including all values into the same variable significantly reduces the amount of links necessary, resulting in lower computing power requirements and a more robust network (Merten, 2004).

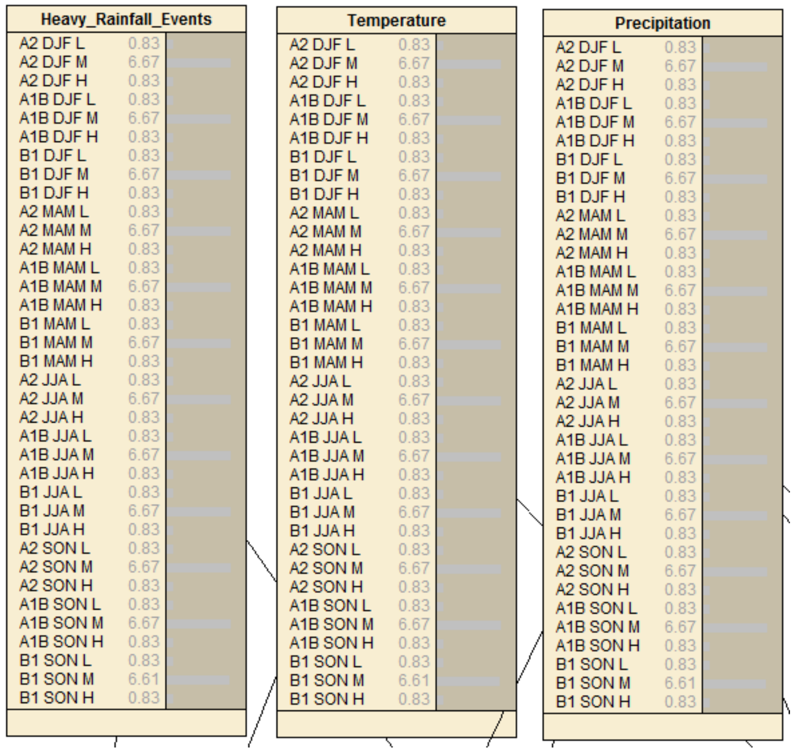

Supplementary Figure 2 – Climate subnet nodes

2.1.4 b) Subnet 2: The impact of future climate upon the site of interest

This subnet contains variables with four different sets of states (Supplementary Table 1). The principle employed is one of selecting as few states as possible, as fewer states result in tackling the Bayesian network's limitation of requiring more nuanced expert judgement for determining the relationship between variables. With basic guidance given by the literature, the layman can more easily define the trend a variable takes according to changes in the parent variable's values (Liu and Wellman, 2002). This subnet's nodes are displayed in Figure 3.

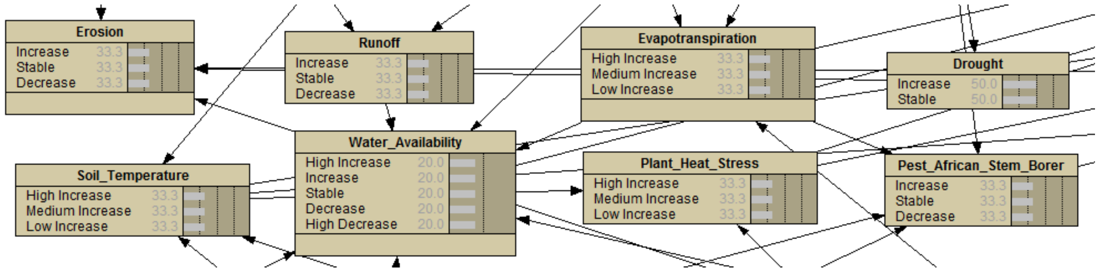

Supplementary Figure 3 – Climate impacts subnet nodes

Variables	States	State Selection Reasoning
Erosion, Runoff, Pests	*Increase, Stable, Decrease*	Three states have been selected to overcome the limitation of the need of expert judgement to define the impact of the climate upon the variable. As indicated in the literature, it is possible for these variables to increase or decrease in the future (IPCC, 2007).
Soil Temperature, Plant Heat Stress, Evapotranspiration	*High Increase, Medium Increase, Low Increase*	These variables are directly linked to the temperature variable in Subnet 1. As temperature values are all increasing, states have been chosen to reflect different levels of increase.
Drought	*Increase, Stable*	As the literature has only described stable or increased drought conditions for Malawi, these states have been selected (IPCC, 2007).
Water Availability	*High Increase, Increase, Stable, Decrease, High Decrease*	The literature on Malawi has indicated that water availability will be one of the biggest factors in future vulnerability (IPCC, 2007). However, the climate data indicates that both reductions and increases are possible (McSweeney, *et al*. 2010). Five states have been selected to account for higher granularity of this variable, while allowing for both potential decrease and increase.

Supplementary Table 1 – Subnet 2 variable description

2.1.4 c) Subnet 3: A description of the characteristics of the site of interest

The states selected within this subnet are taken over directly from the survey results published by the World Bank's "Third Integrated Household Survey" in Malawi (2011). Supplementary Table 2 lists all the states within this subnet, and Supplementary Figure 4 displays its nodes.

Variables	States	State Selection Reasoning	Definition
Waterholding Capacity, Water Use Efficiency	*Good, Medium, Bad*	States selected by	States defined subjectively with

Soil Nitrogen Content	*Good, Fair, Poor*	World Bank (2011) Survey	guidance of World Bank Survey staff by farmer on site.
Soil Organic Carbon, Root Density	*Low, Medium, High*		
Erosion Control, Fertilizer Use	*Yes, No*		
Soil Type	*Sand, Silt, Clay*		
Slope	*Steep, Shallow, Flat*		
Floral Diversity	*Not Diverse, Diverse, Very Diverse*		
Erosion Proneness	*No Erosion, Some Erosion, High Erosion*		
Crop Temperature Sensitivity	*Very Sensitive, Somewhat Sensitive, Moderately Sensitive, Low Sensitivity, Very Low Sensitivity*		

Supplementary Table 2 – Subnet 3 variable description

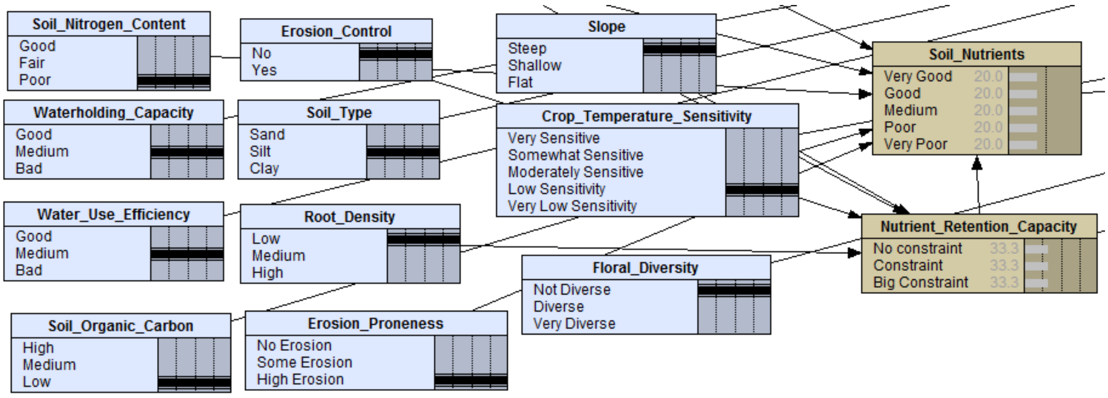

Supplementary Figure 4 – Site characteristics subnet nodes

2.1.4 d) Subnet 4: A set of adaptation options

The adaptation subnet's variables are defined in a binary fashion (Supplementary Figure 5). These two states (*Yes*/*No)* allow for the analysis as described later in section 2.3.

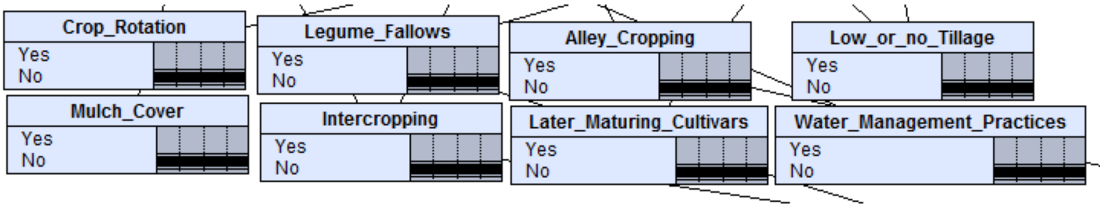

Supplementary Figure 5 – Adaptation options subnet nodes

2.1.5 Link description

A justification will be given as to why links have been defined deterministically or probabilistically. Due to large amount of data that is needed to describe the deterministic relationships between each variable in the network, the link description section found in Appendix B will only list the principles employed in designing these relationships.

2.2 Description of the Bayesian network's output subnet (*Subnet 5*)

The average values of the variables of "*Water Stress*", "*Temperature Stress*" and "*Soil Stress*" define the variable of "*Climate Change Stress*", and the average values of "*Water Resilience, "Temperature Resilience" and "Soil Resilience"* define the variable of "*Climate Change Resilience"*. These variables contain an equal number of states (*Extreme, Very High, High, Medium, Low)*, the values of which range from 1 to 5. These variables bundle the output of previous system variables, and generate data that can be used to analyse overall climate change resilience and stress. These two variables then affect the output-variable of "*Vulnerability of Climate Change*". Supplementary Table 3 describes the states and their attached values, and Supplementary Figure 6 displays the output system.

Vulnerability to Climate Change	
State	Value
Extreme	-4
Very High	-3
High	-2
Somewhat High	-1
Medium	0
Somewhat Low	1
Low	2
Very low	3
No Vulnerability	4

Supplementary Table 3 – *Vulnerability to Climate Change* state value system

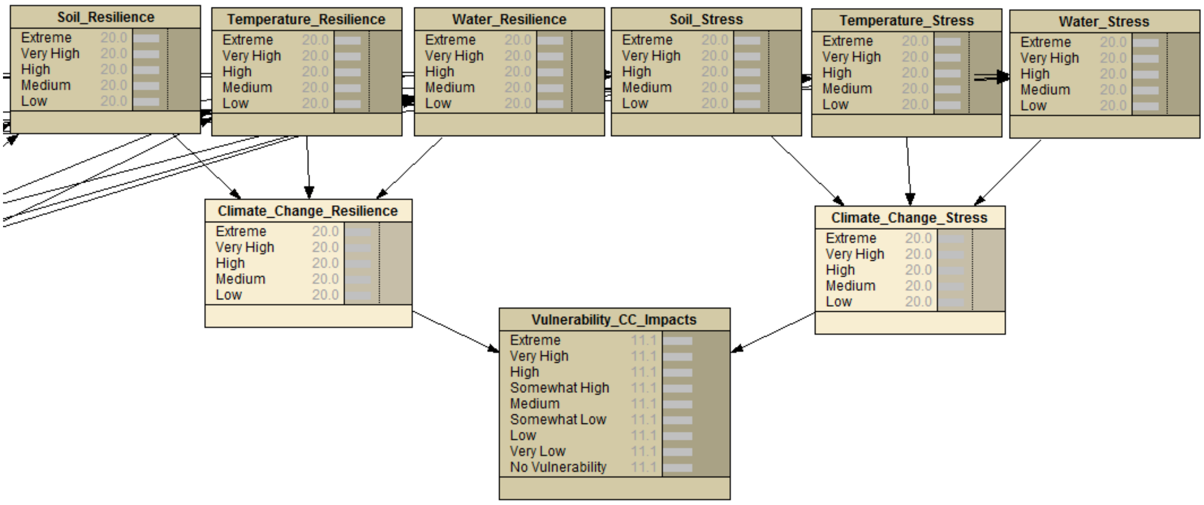

Supplementary Figure 6 – Output system informing *Vulnerability to Climate Change*

Supplementary Figure 7 overleaf shows the entire Bayesian network used.

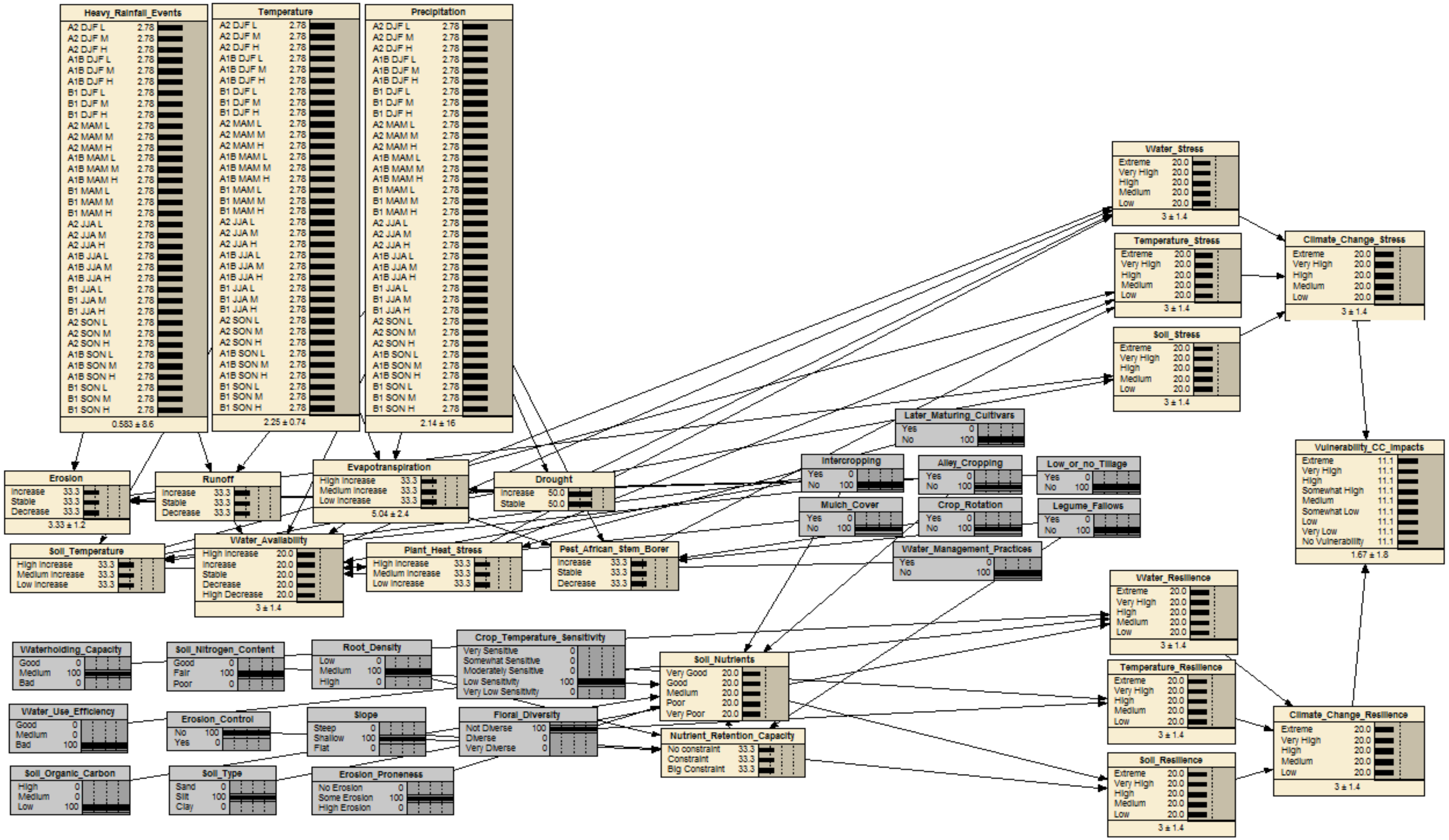

Supplementary Figure 7 – Bayesian network used in this study

Appendix A: Wells' (2012) Framework of Biophysical Indicators (unpublished)

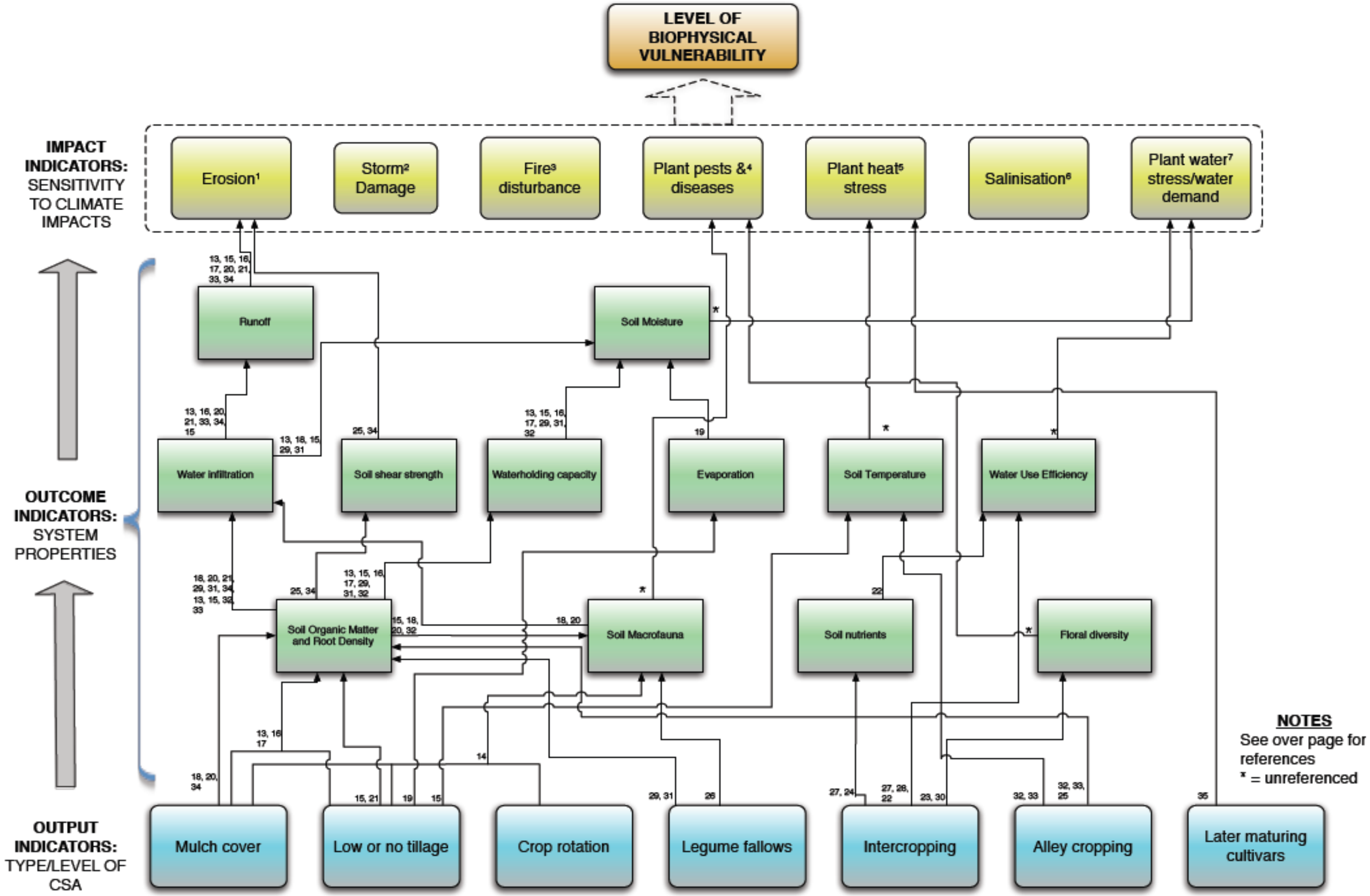

Appendix B – Bayesian Network Links Description

Subnet 1<br>Independent Variable	Dependent Variable(s)	Relationship	Guiding Reference	Unit
Average Temperature (AT)	Evapotranspiration (EV)	If AT ≤ 1, then EV = *Low Increase*	Allen *et al.,* 1998	°C
		If 1 ≤ AT ≤ 2, then EV = *Medium Increase*		°C
		If AT ≥ 3, then EV = *High Increase*		°C
	Soil temperature (ST)	If AT ≤ 1, then ST = *Low Increase*	Zheng *et al.,* 1993	°C
		If 1 ≤ AT≤ 2, then ST = *Medium Increase*		°C
		If AT ≥ 3, then ST = *High Increase*		°C
Average Precipitation (AP)	Runoff (RO)	If AP ≤ -5, then RO = *Decrease*	FAO, 1991	mm/month
		If -5 ≤ AP ≤ 5, then RO = *Stable*		mm/month
		If AP ≥ 5, then RO = *Increase*		mm/month
	Drought (D)	If AP ≤ -15, then D = *Increase*	NDMC, 2013	mm/month
		If AP ≤ -15, then D = *Stable*		mm/month
	Water Availability (WA)	If AP ≤ -15, then DW = *High Decrease*	Kirk, 2007	mm/month
		If -15 ≤ AP ≤ -5, then DW = *Decrease*		mm/month
		If -5 ≤ AP ≤ 5, then DW = *Stable*		mm/month
		If 5 ≤ AP ≤ 15, then DW = *Increase*		mm/month
		If 5 ≤ AP ≤ 15, then DW = *High Increase*		mm/month
	Pest (P)	If AP ≥ 15, then P = *Decrease*	Mailafiya *et al.*, 2011	mm/month
		If 15 ≥ AP ≥ -15, then P = Stable		mm/month
		If AP ≤ -15, the P = *Increase*		mm/month
	Evapotranspiration (EV)	If AP ≤ -15, then EV = *Increase*	Allen et al., 1998	mm/month
		If -15 ≤ AP ≤ 15, then EV = *Medium Increase*		mm/month
		If AP ≥ 15, then EV = *Low Increase*		mm/month
Heavy Rainfall (HR)	Erosion (ER)	If HR ≤ -5, then ER = *Decrease*	Al Kaisi *et al.* 2008	% change
		If -5 ≤ HR ≤ 5, then ER = *Stable*		% change
		If HR ≤ 5, then ER = *Increase*		% change
	Runoff (RO)	If HR ≤ -5, then RO = *Decrease*	Al Kaisi et al. 2008	% change
		If -5 ≤ HR ≤ 5, then RO = *Stable*		% change
		If HR ≤ 5, then RO = *Increase*		% change

**Subnet 2**<br>**Intra-Subnet Relation**	**Dependent Variable(s)**	**Relationship**	**Guiding Reference**
Runoff (RO)	Water Availability (WA)	If RO = *Increase,* then WA = *Increase*<br>If RO = *Stable*, the WA = *Stable*<br>If RO = *Decrease*, then WA = *Decrease*	Al Kaisi *et al*., 2008
Evapotranspiration (EV)	Water Availability (WA)	If EV = *High Increase,* then WA = *High Decrease*<br>If EV = *Medium Increase*, then WA = *Decrease*<br>If EV = *Low Increase,* then WA = *Decrease*	Allen *et al.* 1998
	Pest (P)	If EV = *High Increase*, then P = *Increase*<br>If EV = *Medium Increase*, then P = *Stable*<br>If EV = *Low Increase*, then P = *Stable*	Biovision, 2011
Soil temperature (ST)	Plant Heat Stress (PHS)	If ST = *High Increase*, then PHS = *High Increase*<br>If ST = *Medium Increase*, then PHS = *Medium Increase*<br>If ST = *Low Increase*, then PHS = *Low Increase*	Wang *et al*., 2009
**Inter-Subnet Relation**			
Drought (D)	Water Stress (WS)	If D = *Increase*, then WS = *Extreme*<br>If D = Stable, then WS = *Medium*	/
Plant Heat Stress (PHS)	Water Stress (WS)	If PHS = *High Increase,* then WS = *Very High*<br>If PHS = *Medium Increase*, then WS = *Medium*<br>If PHS = *Low Increase*, then WS = *Low*	Wang *et al*., 2009
	Temperature Stress (TS)	If PHS = *High Increase,* then TS = *Very High*<br>If PHS = *Medium Increase*, then TS = *Medium*<br>If PHS = *Low Increase*, then TS = *Low*	Wang et al., 2009
Water Availability (WA)	Water Stress (WS)	If WA = *High Decrease*, then WS = *Extreme*<br>If WA = *Decrease,* then WS = *Very High*<br>If WA = *Stable,* then WS = *High*<br>If WA = *Increase,* then WS = *Medium*<br>If WA = *High Increase*, then WS = *Low*	/
Evapotranspiration (EV)	Water Stress (WS)	If EV = *High Increase*, then WS = *High*<br>If EV = *Medium Increase*, then WS = *Medium*<br>If EV = *Low Increase*, then WS = *Low*	Allen *et al*., 1998

Subnet 2 (cont'd)<br>Inter-Subnet Relation (cont'd)	Dependent Variable(s)	Relationship	Guiding Reference
Soil Temperature (ST)	Temperature Stress (TS)	If ST = *High Increase,* then TS = *High*	Zheng *et al.*, 1993
		If ST = *Medium Increase*, then TS = *Medium*	
		If ST = *Low Increase,* then TS = *Low*	
	Soil Stress (SS)	If ST = *High Increase,* then SS = *High*	Zheng et al., 1993
		If ST = *Medium Increase*, then SS = *Medium*	
		If ST = *Low Increase,* then SS = *Low*	
Pest (P)	Temperature Stress (TS)	If P = *Increase,* then TS = *High*	NDMC, 2013
		If P = *Stable* then TS = *Medium*	
		If P = *Decrease* then TS = *Low*	
Erosion (E.)	Soil Stress (SS)	If E = *Increase,* then SS = *Extreme*	/
		If E = *Stable* then SS = *Medium*	
		If E = *Decrease* then SS = *Low*	

Subnet 3<br>Intra-Subnet Relation	Dependent Variable(s)	Relationship	Guiding Reference
Soil Nitrogen Content (SNC)	Soil Nutrients (SN)	If SNC = *Good,* then SN = *Good*<br>If SNC = *Fair* then SN = *Medium*<br>If SNC = *Poor* then SN = *Poor*	/
Root Density (RD)	Nutrient Retention Capacity (NRC)	If RD = *High*, then NRC = *No constraint*<br>If RD = *Medium* then NRC = *Constraint*<br>If RD = *Low* then NRC = *Big constraint*	Bierman and Rosen, 2005
Erosion Control (EC)	Nutrient Retention Capacity (NRC)	If EC = *Yes,* then NRC = *No constraint*<br>If EC = *No* then NRC = *Big constraint*	Herbert and Fownes, 1999
Slope (S)	Nutrient Retention Capacity (NRC)	If S = *Steep*, then NRC = *Big constraint*<br>If S = *Shallow,* then NRC = *Constraint*<br>If S = *Flat,* then NRC = *No constraint*	Gyle, 2012
Erosion Proneness (EP)	Soil Nutrients (SN)	If EP = *No Erosion*, then SN = *Good*<br>If EP = *Some Erosion*, then SN = *Medium*<br>If EP = *High Erosion*, then SN = *Poor*	Bierman and Rosen, 2005
Soil Organic Carbon (SOC)	Soil Nutrients (SN)	If SOC = *High*, then SN = *Good*<br>If SOC = *Medium* then SN = *Medium*<br>If SOC = *Low* then SN = *Poor*	Cao *et al.*, 2013
Nutrient Retention Capacity (NRC)	Soil Nutrients (SN)	If NRC = *No constraint,* then SN = *Very Good*<br>If NRC = *Constraint,* then SN = *Medium*<br>If NRC = *Big constraint,* then SN = *Very Poor*	Gyle, 2012
**Inter-Subnet Relation**			
Waterholding Capacity (WHC)	Water Resilience (WR)	If WHC = *Good*, then WR = *High*<br>If WHC = *Medium* then WR = *Medium*<br>If WHC = *Bad* then WR = *Low*	/
Crop Temperature Sensitivity (CTS)	Temperature Resilience (TR)	If CTS = *Very Sensitive*, then TR = *Extreme*<br>If CTS = *Somewhat Sensitive*, then TR = *Very High*<br>If CTS = *Moderately Sensitive*, then TR = *High*<br>If CTS = *Low Sensitivity*, then TR = *Medium*<br>If CTS = *Very Low Sensitivity*, then TR = *Low*	Crafts-Brandner and Salvucci, 2002

**Subnet 3 (cont'd)**<br>**Inter-Subnet Relation (cont'd)**	**Dependent Variable(s)**	**Relationship**	**Guiding Reference**
Floral Diversity (FD)	Temperature Resilience (TR)	If FD = *Very Diverse*, then TR = *High*	Wells, 2012 (Appendix A)
		If FD = *Diverse*, then TR = *Medium*	
		If FD = *Not Diverse*, then TR = *Low*	
Soil Type (ST)	Water Resilience (WR)	If ST = *Sand*, then WR = *Low*	Kramer and Boyer 1995
		If ST = *Silt* then WR = *Medium*	
		If ST = *Clay* then WR = *High*	
Soil Nutrients (SN)	Soil Resilience (SR)	If SN = *Very Good,* then SR = *Extreme*	/
		If SN = *Good,* then SR = *Very High*	
		If SN = *Medium,* then SR = *High*	
		If SN = *Poor,* then SR = *Medium*	
		If SN = *Very Poor* then SR = *Low*	
Nutrient Retention Capacity (NRC)	Soil Resilience (SR)	If NRC = *No constraint*, then SR = *High*	Gyle, 2012
		If NRC = *Constraint*, then SR = *High*	
		If NRC = *Big constraint*, then SR = *High*	

**Subnet 4**<br>**Independent Variable**	Dependent Variable(s)	Relationship	Guiding Reference
Mulch Cover (MS)	Water Availability (WA)	If MS = *Yes,* then WA = +1<br>If MS = *No,* then nothing	Boli *et al.* 1993
Low/No Tillage (LNT)	Evapotranspiration (E.)	If LNT = *Yes* then E = +1<br>If LNT = *No,* then nothing	Lal, 1974
Crop Rotation (CR)	Pest (P)	If CR = *Yes,* then P = +1<br>If CR = *No,* then nothing	Tierfelder and Wall, 2009
Intercropping (I)	Water Availability (WA)	If I = *Yes,* then WA = +1<br>If I = *No,* then nothing	Chirva *et al.* 2007
Legume Fallows (LF)	Pest (P)	If LF = Yes then P = +1<br>If LF = *No,* then nothing	Sileshi and Mafongoya, 2006
Alley Cropping (AC)	Soil Nutrients (SN)	If AC = *Yes* then SN = +1<br>If AC = *No,* then nothing	Hulugalle and Kang, 1990
Later Maturing Cultivars (LMC)	Plant Heat Stress (PHS)	If LMC = *Yes* then PHS = +1<br>If LMC = *No,* then nothing	Tigem *et al*. 2009
Water Management Practices (WMP)	Water Availability (WA)	If WMP = *No,* then WA = +1<br>If WMP = *No,* then nothing	/

Appendix C – References

Al Kaisi M., Helmers M. (2008) "*Heavy Rain, Soil Erosion and Nutrient Losses*" [online] Available at: http://www.extension.iastate.edu/CropNews/2008/0604MAlKaisiMHelmers.htm [Accessed: 20/8/2013]

Allen R.G., Pereira L.S., Raes D., Smith M. (1998) "*Crop evapotranspiration – Guidelines for computing crop water requirement – FAO Irrigation and drainage paper 56*" [online] Available at: http://www.engr.scu.edu/~emaurer/classes/ceng140_watres/handouts/FAO_56_Evapotranspiration.pdf [Accessed: 20/8/2013]

Banda A., Maghembe D., Ngugi D. "Effect of intercropping maize and closely spaced Leucaena hedgerows on soil conservation and maize yield on a steep slope at Ntcheu, Malawi" in *Agricforestry Systems*, vol.27, pp.17-22

Bierman P. M., Rosen C.J. (2005) "*Nutrient Cycling & Maintaining Soil Fertility in Fruit and Vegetable Systems*" [online] Available at: http://www.extension.umn.edu/distribution/horticulture/m1193.html [Accessed: 20/8/2013]

Biovision (2011) "*African Maize Stalkborer*" [online] Available at: http://www.infonet-biovision.org/default/ct/102/pests [Accessed: 3/7/2013]

Boli *et al.* (1993) in Roose E., Barth B. (2001) "Organic matter management for soil conservation and productivity restoration in Africa: a contribution from Francophone research" in *Nutrient Cycling in Agroecosystems*, vol.61, pp.159-170

Cao Y., Wang X., Lu X., Yan Y., Fan J. (2013) "Soil organic carbon and nutrients along an alpine grassland transect across Northern Tibet" in *Journal of Mountain Science*, vol.10(4), pp.564-573

Chirva P.W., Ong C.K., Maghembe J., Black C.R. (2006) "Soil water dynamics in cropping systems containing Gliricidia sepium, pigeonpea and maize in southern Malawi" in *Agroforestry Systems*, vol.69, pp.29-43

Crafts-Brandner S.J., Salvucci M.E. (2002) "Sensitivity of Photosynthesis in a C4 Plant, Maize, to Heat Stress" in *Plant Physiology*, vol.129(4), pp.1773-1780

Druzdzel M.J., Unisko A. (2008) "*The Impact of Overconfidence on Practical Accuracy of Bayesian Network Models: An Empirical Study*" [online] Available at: ftp://ftp.pitt.edu/users/d/r/druzdzel/uai08.pdf [Accessed: 3/7/2013]

Easterling W.E., Agarval P. (2007) "Food, Fibre and Forest Products" in *Climate Change 2007: Impacts, Adaptation and Vulnerability. Contribution of Working Group II to the Fourth Assessment Report of the Intergovernmental Panel on Climate Change*, Parry K.L., Canziani

O.F., Palutikof J.P., van der Linden P.J., Hanson C.E., (eds.), p. 284, Cambridge University Press, Cambridge

Environmental Protection Agency, United States (EPA) (2013) "*Agriculture and Food Supply Impacts and Adaptation*" [online] Available at: http://www.epa.gov/climatechange/impacts-adaptation/agriculture.html#impactscrops [Accessed: 30/6/2013]

Food and Agriculture Organisation (FAO) (1991) "*Rainfall-runoff analysis*" [online] Available at: http://www.fao.org/docrep/u3160e/u3160e05.htm [Accessed: 20/8/2013]

Food and Agriculture Organization (FAO) (2006) "*Malawi*" [online] Available at: http://www.fao.org/nr/water/aquastat/countries_regions/malawi/index.stm [Accessed: 3/7/2013]

Garba M., Renard C. (1991) in *Proceedings of the International Association of Hydrological Sciences Niamey Workshop, February 1991*, (International Association of Hydrological Sciences)

Gyle Y. (2012) "*Nutrient Retention model: Invest 2.2.1*" [online] Available at: http://ncp-dev.stanford.edu/~dataportal/training_feb2012_stanford/InVEST_Nutrient%20model.pdf [Accessed: 20/8/2013]

Hemp A. (2006) "The Banana Forests of Kilimanjaro: Biodiversity and Conservation of the Chagga Homegardens", *Biodiversity and Conservation,* vol.15, pp.1193-1217

Herbert D.A., Fownes J.H. (1999) "Forest Productivity and Efficiency of Resource Use Across a Chronosequence of Tropical Montane Soils" in *Ecosystems*, vol.2(3), pp.242-254

Huang W., Luukkanen O., Johanson S. (2002) "Agroforestry for biodiversity conservation of nature reserves: functional group identification and analysis", *Agroforestry Systems,* vol.55, pp.65-72

Hulugalle N.R., Kang B.T. (1990) "Effect of hedgerow species in alley cropping systems on surface soil physical properties of an Oxic Paleustalf in south-western Nigeria" in *The Journal of Agricultural Science,* vol.114, p.301

Hulugalle N., Ndi J. (1993) "Effects of no-tillage and alley cropping on soil properties and crop yields in a Typic Kandiudult of southern Cameroon", *Agroforestry Systems,* vol.22, pp.207-220

Intergovernmental Panel on Climate Change (IPCC) (2007) "*The Fourth Assessment Report of the Intergovernmental Panel on Climate Change*" [online] Available at: http://www.ipcc.ch/publications_and_data/ar4/syr/en/contents.html [Accessed: 22/5/2013]

Jackson N., Wallace J., Ong C. (2000) "Tree pruning as a means of controlling water use in an agroforestry system in Kenya", *Forest Ecology and Management,* vol.126, pp.133-148

Johnson S. (2009) "Integrated Bayesian Network frameworks for modelling complex ecological issues", *Thesis Paper submitted for the degree of Doctor of Philosophy*, *Queensland University of Technology* [online] Available at: http://eprints.qut.edu.au/32002/1/Sandra_Johnson_Thesis.pdf [Accessed: 3/7/2013]

Kirk, T. (2007) "Precipitation and its Effect on Groundwater Supply in WRD's Region" in *WRD Technical Bulletin*, vol.11 [online] Available at: http://www.wrd.org/engineering/reports/TB11_Spring_2007_Precipitation.pdf [Accessed: 20/8/2013]

Kramer P.J., Boyer J.S. (1995) "*Water relations of plants and soils*" Waltham: Academic Press Inc.

Rishirumuhirwa (1997) in E. Roose, B. Barth, Organic matter management for soil conservation and productivity restoration in Africa: a contribution from Francophone research, pp.159-170 (2001)

Lal R., (1974) "No-tillage effects on soil properties and maize production is western Nigeria" in *Plant and Soil*, vol.331, pp.321-331

Letourneau D.K., Armbrecht I., Salguero Rivera B., Montoya Lerma J., Jiménez Carmona E., Constanza Daza M., Escobar S., Galindo V., Gutiérrez C., Duque López S., López Mejía J., Maritza Acosta Rangel A., Herrera Rangel J., Rivera L., Arturo Saavedra C., Marina Torres A., Reyes Trujillo A. (2011) "Does plant diversity benefit agroecosystems? A synthetic review" in *Ecological Applications*, vol.21, pp.9-21

Liu C., Wellman M.P. (2002) "Evaluation of Bayesian Networks with flexible state-space abstraction methods" in *International Journal of Approximate Reasoning*, vol.30, pp.1-39

Mailafiya D.M., Le Ru B.P., Waitherero Kairu E., Dupas S., Calatayud P. (2011) "Paratsitism of Lepidopterous Stem Borers in Cultivated and Natural Habitats" in *Journal of Insect Science*, vol.11, pp.1-15

McSweeney C., New M., Lizcano G. (2010) "*UNDP Climate Change Country Profiles – Malawi*" [online] Available at: http://www.geog.ox.ac.uk/research/climate/projects/undp-cp/index.html?country=Malawi&d1=Reports [Accessed: 25/5/2013]

Merten C. (2004) "*Incremental Compilation of Object-Oriented Bayesian Networks*" [online] Available at: http://www.cs.ubc.ca/~murphyk/Teaching/CS532c_Fall04/Projects/merten.pdf [Accessed: 3/7/2013]

Mueller C., Cramer W., Hare W.L., Lotze-Campen H. (2011) "Climate change risks for African agriculture" in *Proceedings of the National Academy of Sciences of the United States of America*, March 15, pp.4313-4315

Musango J.K., Peter C. (2011) "A Bayesian approach towards facilitating climate change adaptation research on the South African agricultural sector" *Agrekon.* **46(2)**, 245-259

National Drought Mitigation Centre (NDMC) (2013) "*What is Drought?*" [online] Available at: http://drought.unl.edu/DroughtBasics/WhatisDrought.aspx [Accessed 8/7/2013]

Neil M., Fenton N., Nielson L. (2000) "Building large-scale Bayesian Networks" in *The Knowledge Engineering Review*, vol. 15(3), pp.257-284

Nhamo (2007) in C. Thierfelder, P. C. Wall, in *Innovations as Key to the Green Revolution in Africa*, A. Bationo, B. Waswa, J. M. Okeyo, F. Maina, J. M. Kihara, Eds. (Springer Netherlands, Dordrecht, 2011)

Norsys (2013b) "*Node Kind*" [online] Available at: http://www.norsys.com/WebHelp/NETICA/X_Node_Kind.htm [Accessed: 30/6/2013]

Obi M.E., Nnabude P.C. (1988) "The Effects of Different Management Practices on the Physical Properties of a Sandy Laom Soil in Southern Nigeria" in *Soil and Tillage Research*, vol.12, pp.81-90

Ohiri A., Ezumah H.C. (1990) "Tillage effects on cassava (Manihot esculenta) production and some soil properties", *Soil and Tillage Research,* vol.17, pp.221-229

Opara-Nadi O.A., Lal, R. (1987) "Effects of No-Till on Disc Plowing With and Without Residue Mulch on Tropical Root Crops on Southeastern Nigeria", *Soil and Tillage Research*", vol.9 pp.231-240

Phiri E., Verplancke H., Kwesiga F., Mafongoya P. (2003) "Water balance and maize yield following improved sesbania fallow in eastern Zambia", *Agroforestry Systems,* vol.59, pp.197-205

Rishirumuhirva (1997) in Roose E., Barth B. (2001) "Organic matter management for soil conservation and productivity restoration in Africa: a contribution from Francophone research" in *Nutrient Cycling in Agroecosystems*, vol.61, pp.159-170

Saka A.R., Bunderson W.T., Itimu O.A., Phombeya H.S.K., Mbekeani Y. (1994)"The effects of Acacia albida on soils and maize grain yields under smallholder farm conditions in Malawi", *Forest Ecology and Management,* vol.64, pp.217-230

Sileshi G., Mafongoya P.L. (2006) "Long-term effects of improved legume fallows on soil invertebrate macrofauna and maize yield in eastern Zambia" in *Agriculture, Ecosystems & Environment*, vol.115, pp.69-78

Sinclair T.R., Tanner C.B., Bennet J.M. (1984) "*Water-Use Efficiency in Crop Production*" in *BioScience*, vol.34(1), pp.36-40

Thierfelder C., Wall P.C. (2009) "Effects of conservation agriculture techniques on infiltration and soil water content in Zambia and Zimbabwe", in *Soil and Tillage Research*, vol.105, pp.217-227

Tingem M., Rivington M., Bellocchi G. (2009) "Adaptation assessments for crop production in response to climate change in Cameroon" in *Agronomy for Sustainable Development* vol.29, pp.247-256

Torquebiau E.F., Kwesiga F. (1996) "Root development in a Sesbania sesban fallow-maize system in Eastern Zambia", *Agroforestry Systems,* vol.34, pp.193-211

Utomo and Guritno (1984) in R. H. Howeler, H. C. Ezumah, D. J. Midmore, "Tillage systems for root and tuber crops in the tropics", *Soil and Tillage Research,* vol. 27, pp.211-240

Wang E., Vinocur B., Altman A. (2003) "Plant responses to drought, salinity and extreme temperatures: towards genetic engineering for stress tolerance" in *Planta*, vol.218(1), pp.1-14

World Bank (2011) "*Data – Malawi: Third Integrated Household Survey*" [online] Available at: http://web.worldbank.org/WBSITE/EXTERNAL/EXTDEC/EXTRESEARCH/EXTLSMS/EXTSURAGRI/0,,contentMDK:23156208~pagePK:64168445~piPK:64168309~theSitePK:7420261,00.html [Accessed: 2/7/2013]

Zheng D., Hunt E.R., Running S.W. (1993) "A daily soil temperature model based on air temperature and precipitation for continental applications" in *Climate Research*, vol.2, pp.183-191
